# Supplementary material for: Radiation-induced DNA damage and repair effects on 3D genome organization
Source: Nat Commun. 2020 Dec 2;11:6178. doi: 10.1038/s41467-020-20047-w (PMC7710719; doi:10.1038/s41467-020-20047-w)
Supplement: Supplementary file 1 — Supplementary Information [file 41467_2020_20047_MOESM1_ESM.pdf]

a

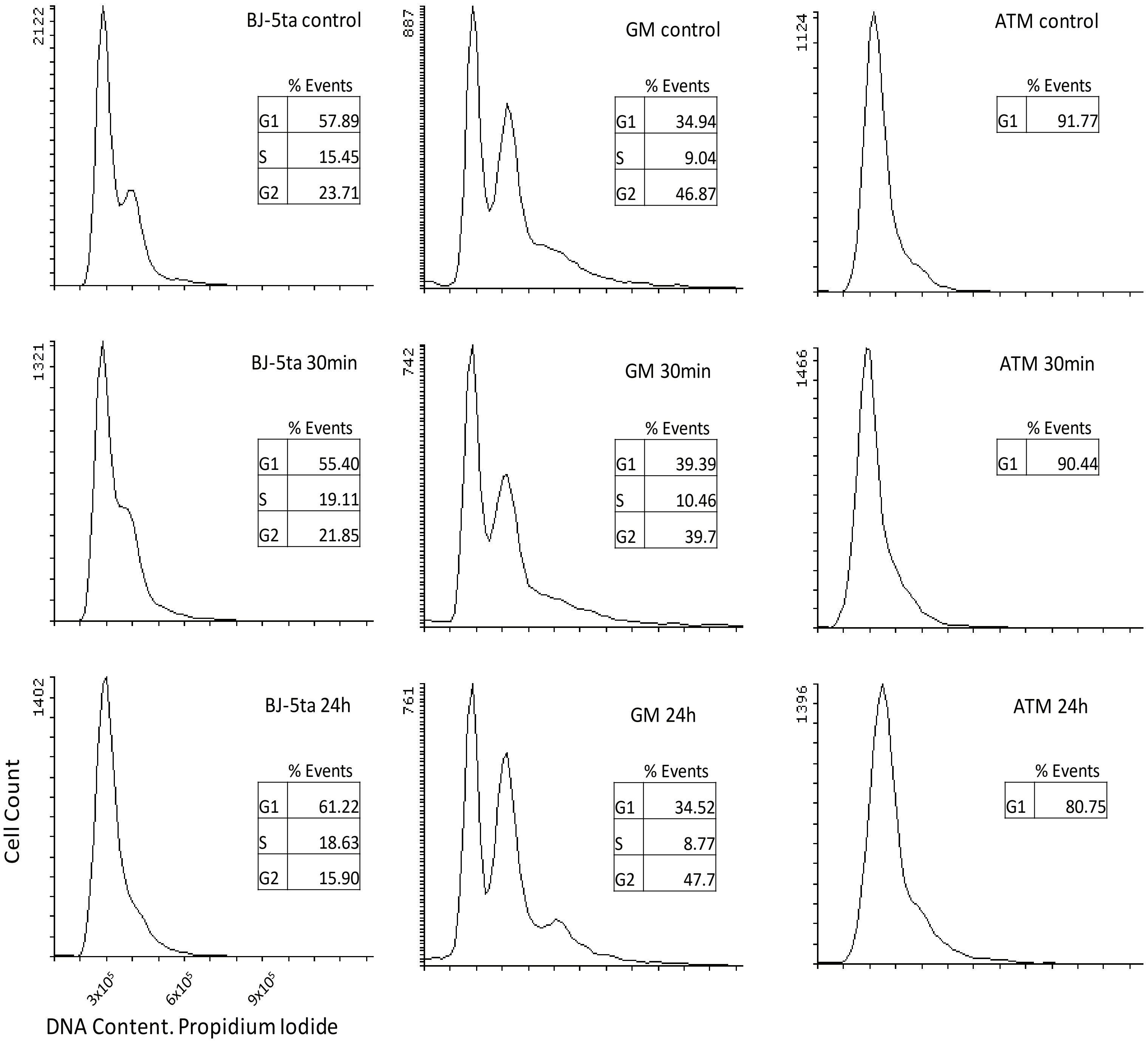

Supplementary Figure 1. a Flow cytometry analysis of BJ-5ta, GM12878, and ATM-hTERT cells before and after irradiation. Y-axis shows cell counts while x-axis represents the fluorescence measurement of DNA content. BJ-5ta cells are predominantly in G1 in all conditions but contain a small population of G2 in control and 30 minutes post IR. GM12878 cells maintain a very similar cell cycle state distribution before and after IR. ATM-hTERT cells are mostly G1 stalled in all conditions.

b

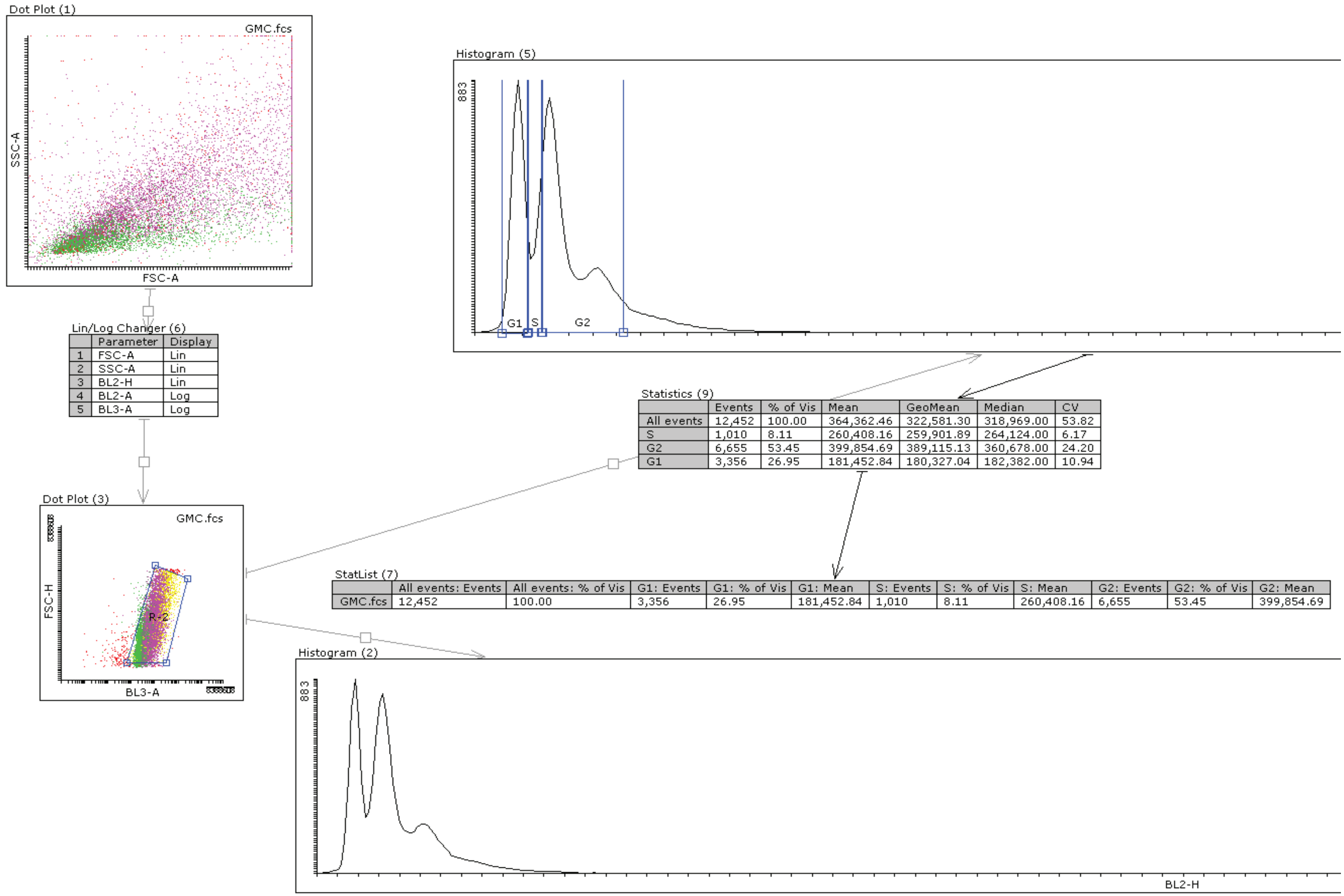

Supplementary Figure 1b. Flow gating strategy. Colors on all scatterplots reflect the final classification as G1/S/G2. The top left plot (FSC vs. SSC) shows the total population of measured events. Then gate P-2 on the FSC-H vs. BL3-A (viability dye) plot is used to exclude doublets and small particulates. Gated viable single cells are then plotted in a histogram along the BL2-H axis (propidium iodide for DNA content) and the gating for determining G1, S, and G2 classifications is shown.

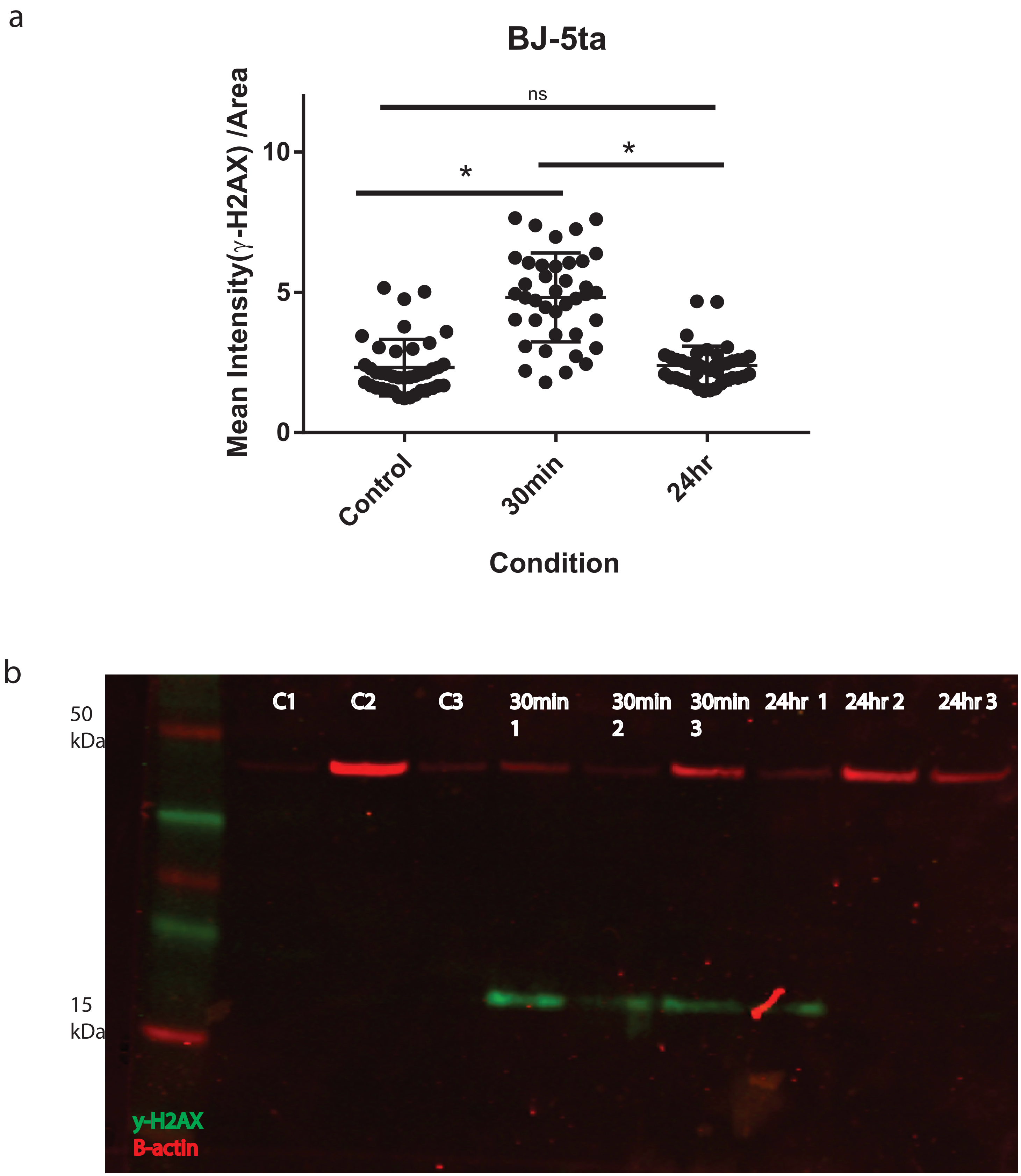

Supplementary Figure 2. γH2AX levels increase in BJ-5ta after exposure to IR. **a** Quantification of mean γH2AX fluorescence intensity per nucleus area in BJ-5ta cells after exposure to IR (n=40 cells, * p = <0.0001, one way ANOVA with Tukey's multiple comparison test. Data is presented as the mean of SD with the 25th (bottom line) and 75th (Top line) percentile shown). **b** Western blot for β-actin (red) and γH2AX (green). Bands around ~15 kDa indicate the presence of γH2AX.

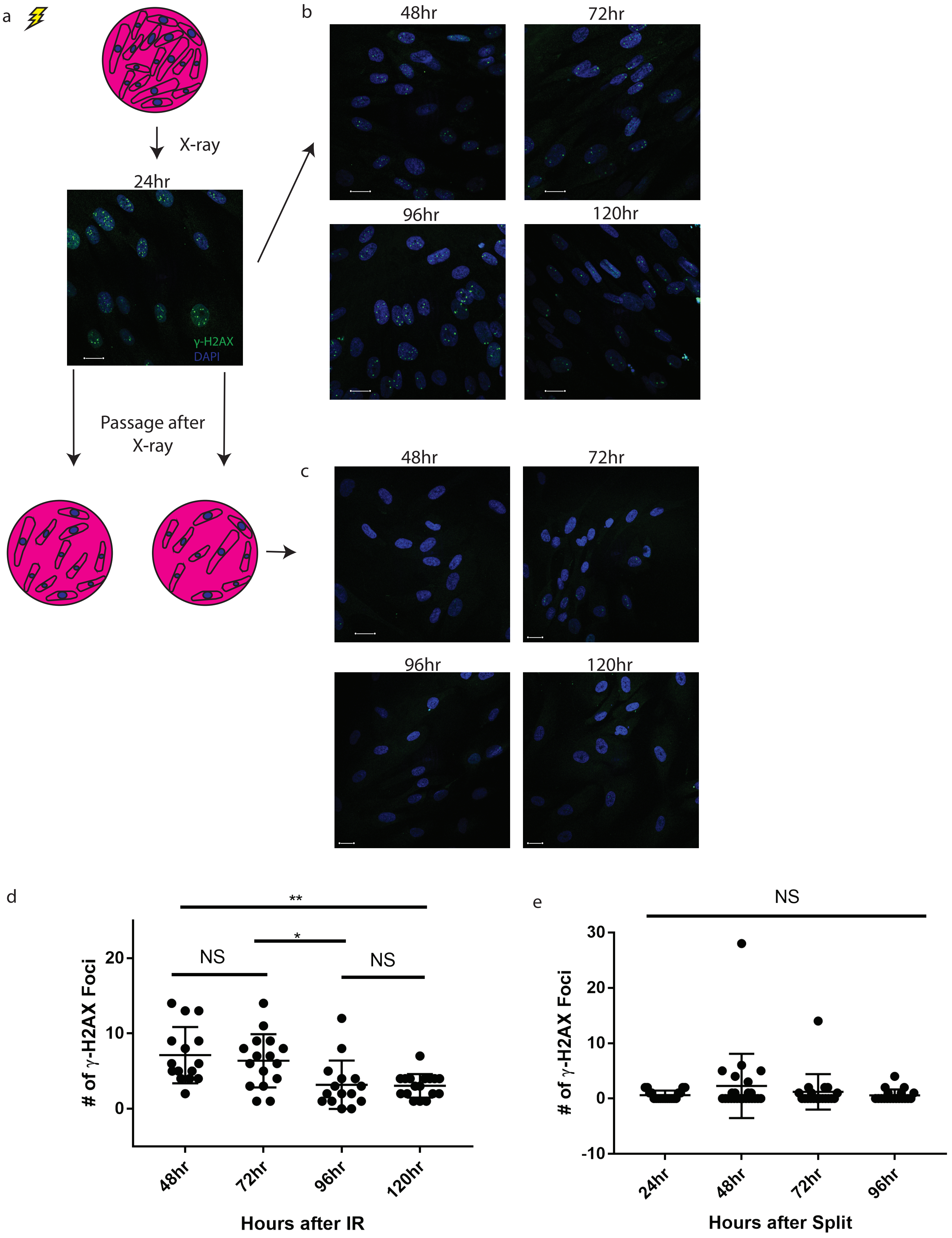

Supplementary Figure 3. Initially unrepaired γH2AX foci remain until after passaging BJ-5ta cells. **a** Schematic of experimental procedure. BJ-5ta were irradiated with 5 Gy X-rays and then grown for up to 120 hours in the same dish (b) or passaged 24 hours after irradiation and split into separate dishes and grown for up to 120 hours (c). **b** BJ-5ta stained with γH2AX (green) and DAPI (blue) after exposure to 5 Gy X-rays in a confluent contact-inhibited culture. Scale bar: 20 µm. **c** BJ-5ta stained with γH2AX (green) and DAPI (blue) in cultures passaged 24 h after exposure to 5 Gy X-rays and then allowed to continue growing for indicated total times after X-ray. Scale bar: 20 µ m. **d** Quantification of γH2AX foci count per nucleus for confluent culture over time (as in b) (n=15 cells, * $p<0.02$** $p<0.005$, one-way ANOVA. Data is presented as the mean of SD with the 25th (bottom line) and 75th (Top line) percentile shown). **e** Quantification of γH2AX foci count per nucleus for cells passaged after exposure to X-ray (as in c). Time reports time after passaging (so 24 h after split = 48 h total after Xray) (n=19 cells for 24hr, 72hr, and 96hr. n=24 cells for 48hr, one way ANOVA. Data is presented as the mean of SD with the 25th (bottom line) and 75th (Top line) percentile shown).

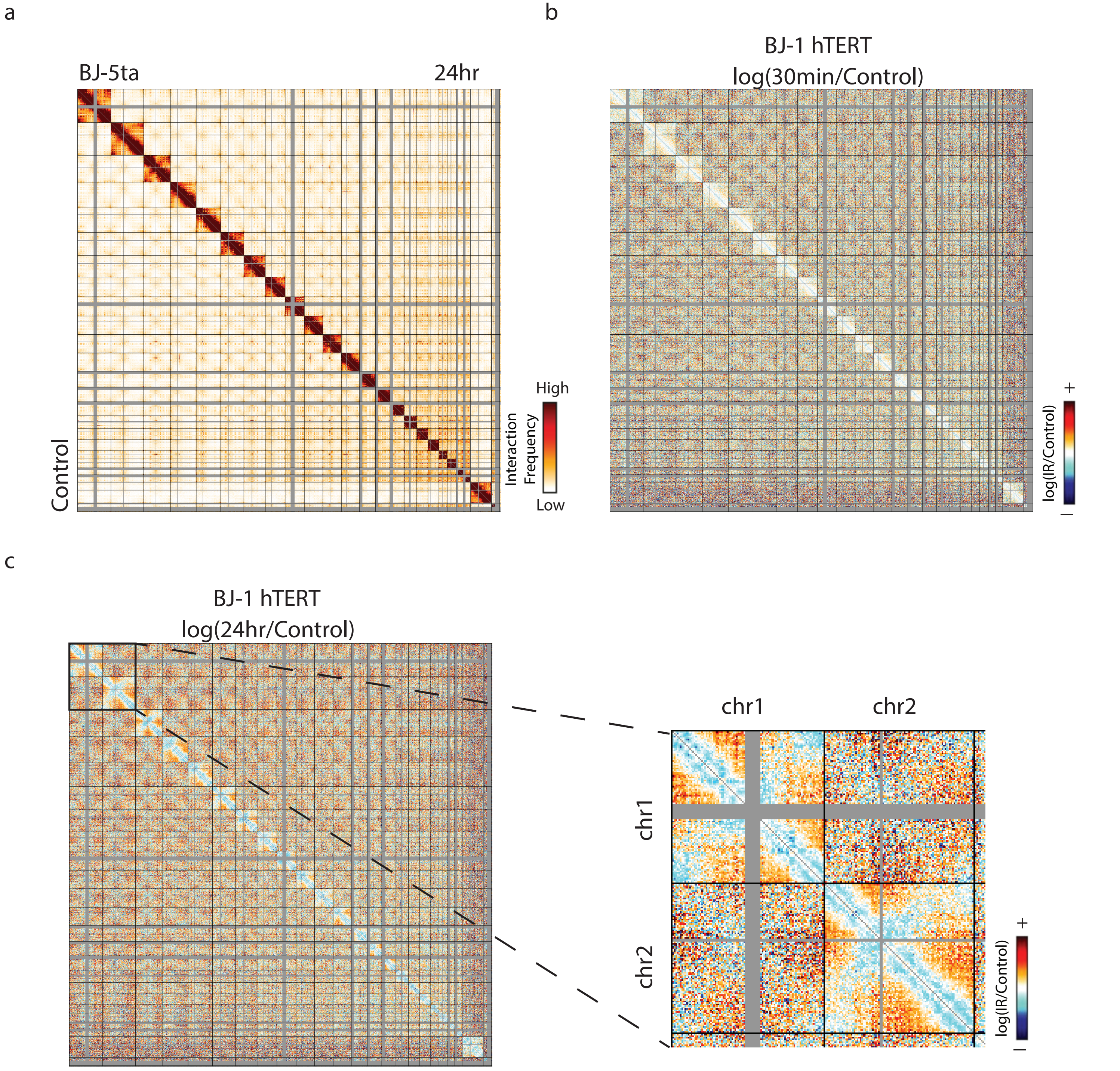

Supplementary Figure 4. BJ-5ta and BJ1-hTERT share similar responses to IR. **a** Genome wide contact map of non-irradiated BJ-5ta (left) and 24 hours after irradiation (right). **b** log2(30 minutes post IR/Control) BJ1-hTERT contact heatmap in 2.5 Mb bins. **c** log2(24 hours post IR/Control) BJ1-hTERT contact heatmap in 2.5 Mb bins. Inset reveals loss of interactions between telomeres after IR in chromosomes 1 and 2 analogous to Fig. 1d.

a

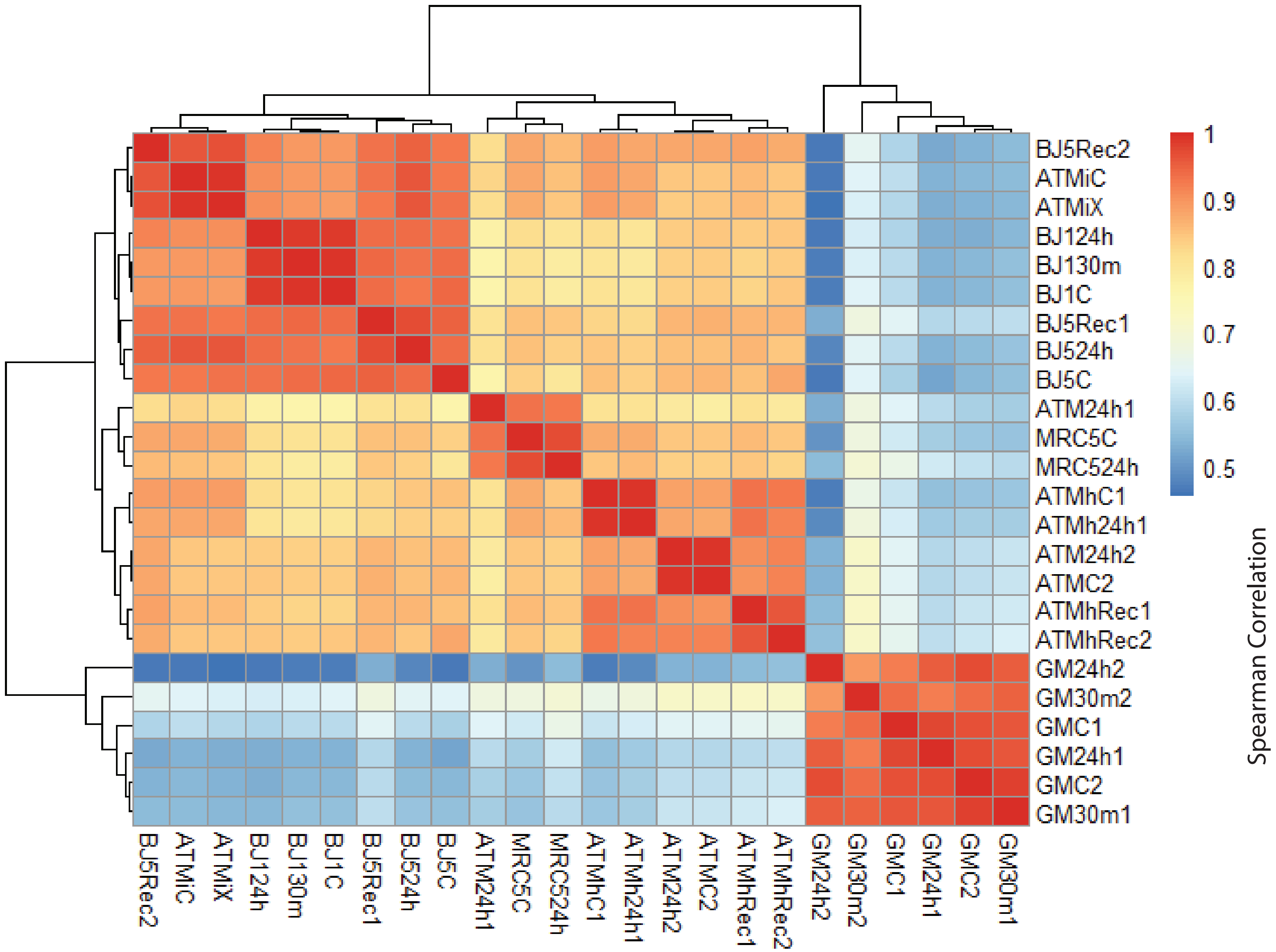

Supplementary Figure 5. Reproducibility correlation plot for all Hi-C experiments. **a** Spearman correlation-based reproducibility is calculated as described in the Methods. ATM proficient fibroblasts mostly cluster together and separately from ATM deficient fibroblasts. GM12878 cells cluster separately from fibroblasts. C= Control, X=Xray, Rec=5 day recovery, 1 and 2 = replicate numbers, ATMi = ATM inhibitor treatment of BJ5-ta, ATMh = ATM mutant cells.

a

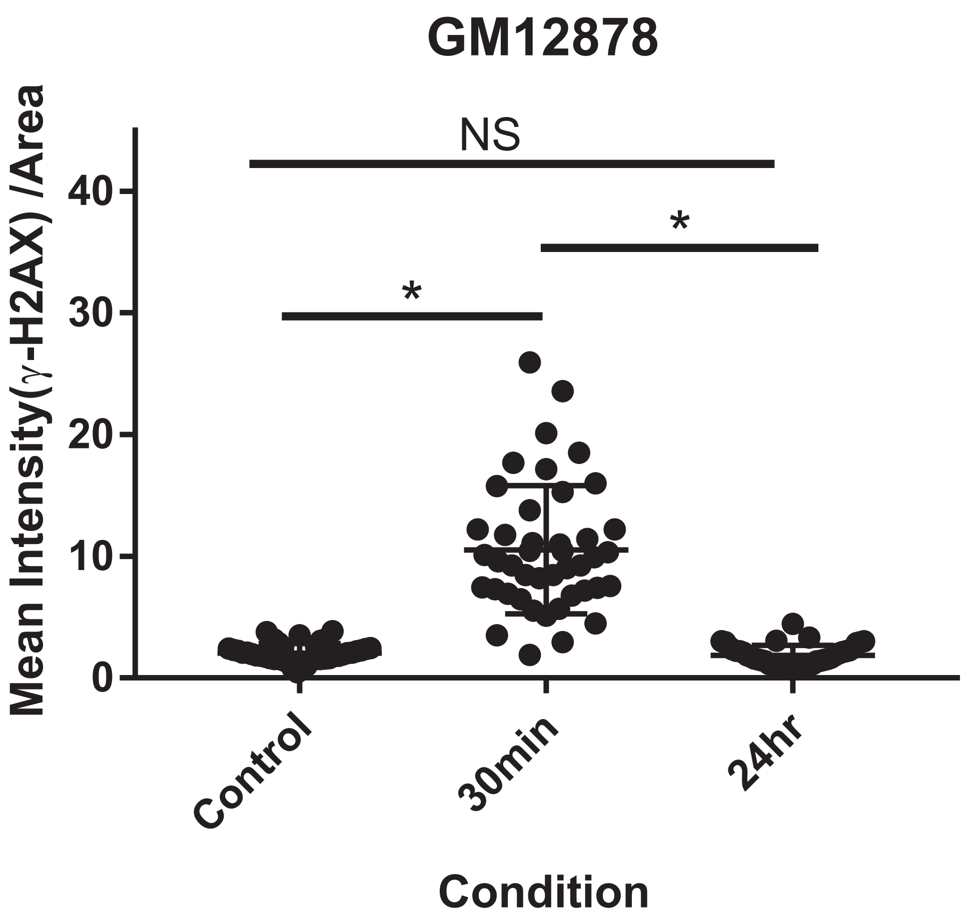

b

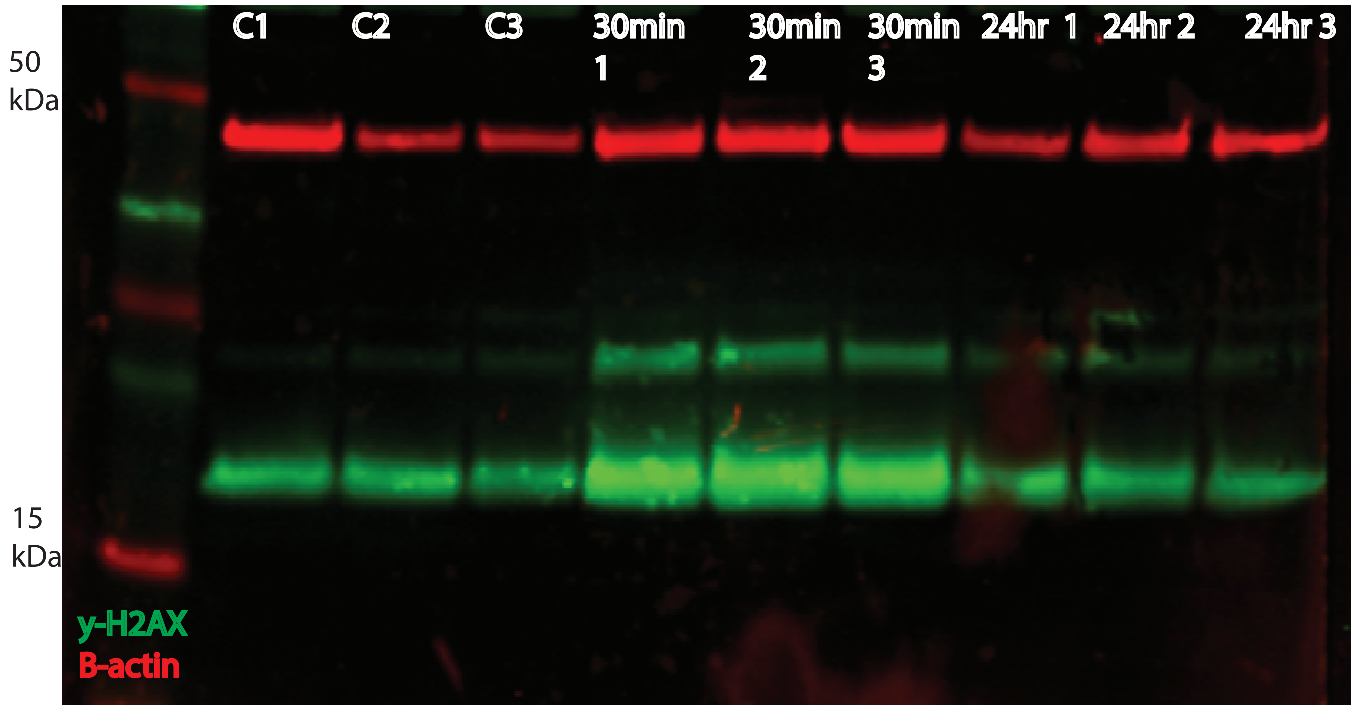

Supplementary Figure 6. γH2AX levels increase in GM12878 after exposure to IR. **a** Quantification of mean γH2AX fluorescence intensity per nucleus area in GM12878 cells after exposure to IR (n=40 cells, * p = 0.0001, one-way ANOVA. Data is presented as the mean of SD with the 25th (bottom line) and 75th (Top line) percentile shown). **b** Western blot analyses for β-actin (red) and γH2AX (green). Bands around ~15 kDa indicate the presence of γH2AX. GM12878 contains more γH2AX in control cells than BJ-5ta, but this may be due to ongoing cell division, including replication stress.

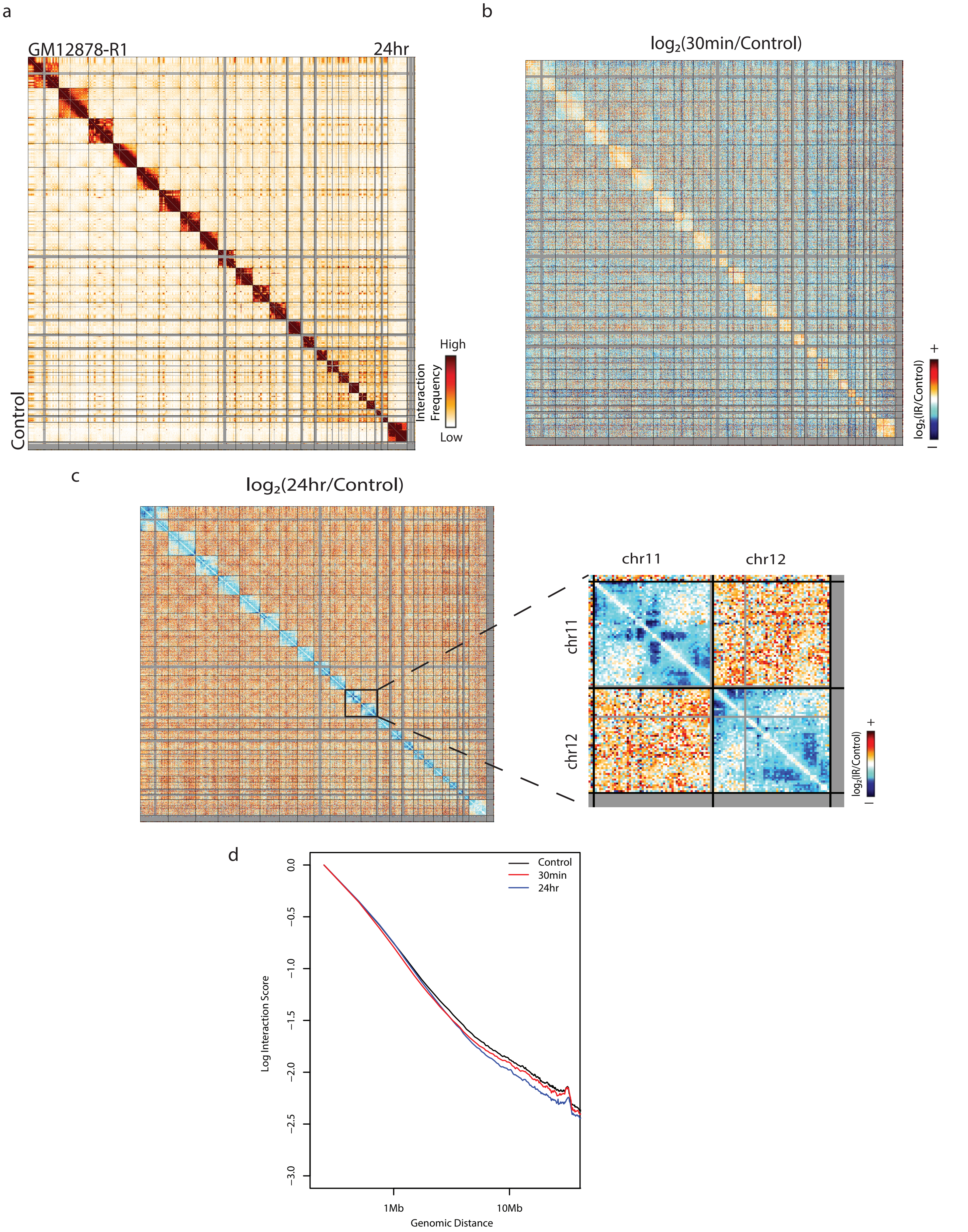

Supplementary Figure 7. Similar major features of GM12878 genome structure change after IR are observed in a biological replicate (R1 here, R2 shown in main text figures). **a** Genome wide contact map of non-irradiated GM12878 (bottom left) and 24 hours after irradiation (upper right). **b** log2(30 minutes post IR/Control) GM12878 contact heat-map in 2.5 Mb bins. **c** log2(24 hours post IR/Control) GM12878 contact heatmap in 2.5 Mb bins. Inset reveals similar specific changes within chr11 and chr12 here in Replicate 1 as shown in main Figure 2 (Replicate 2). **d** Scaling plots showing average decay of contacts with distance across all chromosomes at a 250 kb bin size for GM12878 (R1) before and after IR.

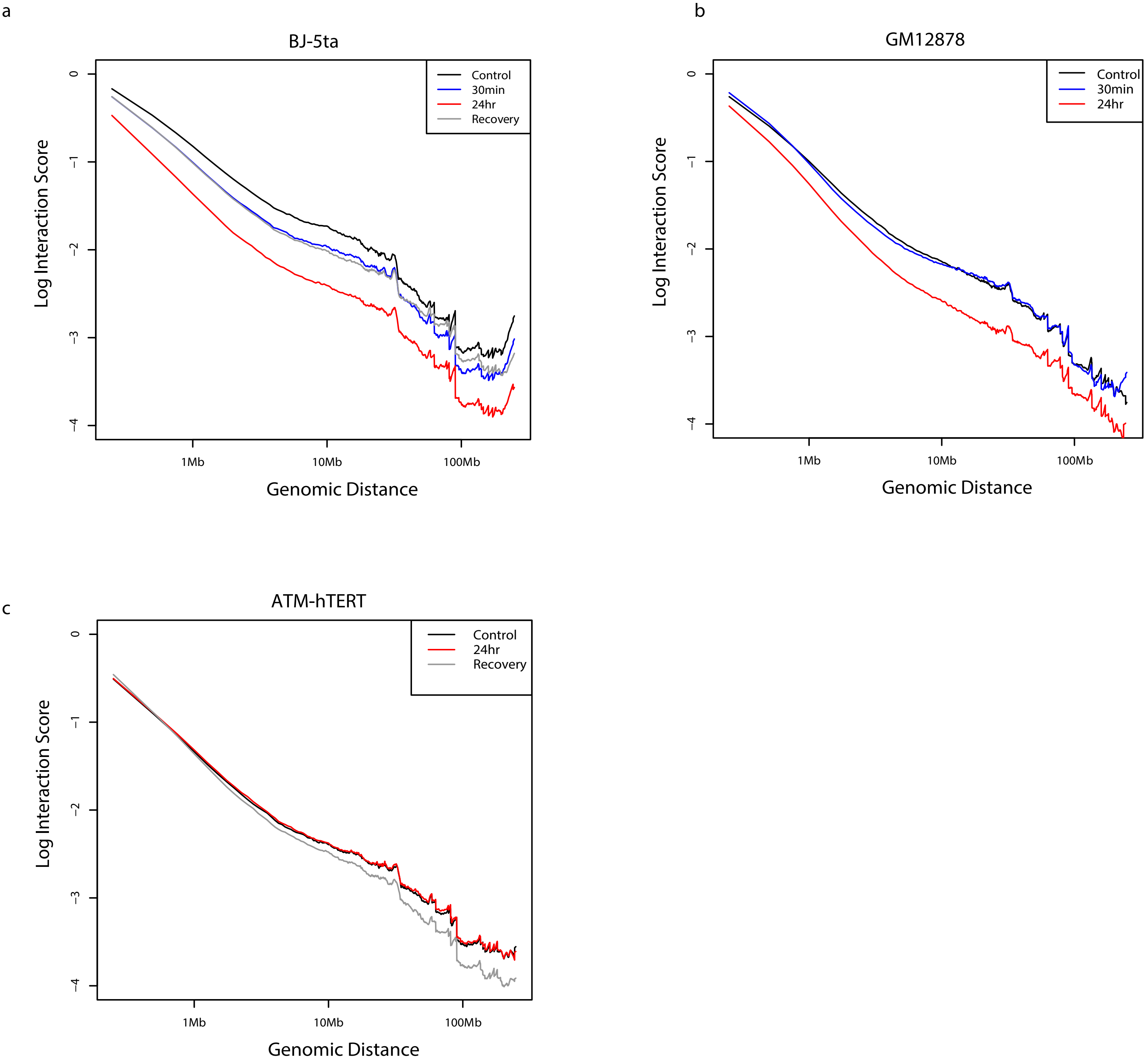

Supplementary Figure 8. Scaling plots showing average decay of contacts with distance across all chromosomes at a 250 kb bin size without normalizing to the same starting contact value for **(a)** BJ-5ta **(b)** GM12878 (R2) or **(c)** ATM mutant cells for control (pre-irradiation), 30 minutes post IR, 24 hours post IR, or 5 days post IR (“Recovery”).

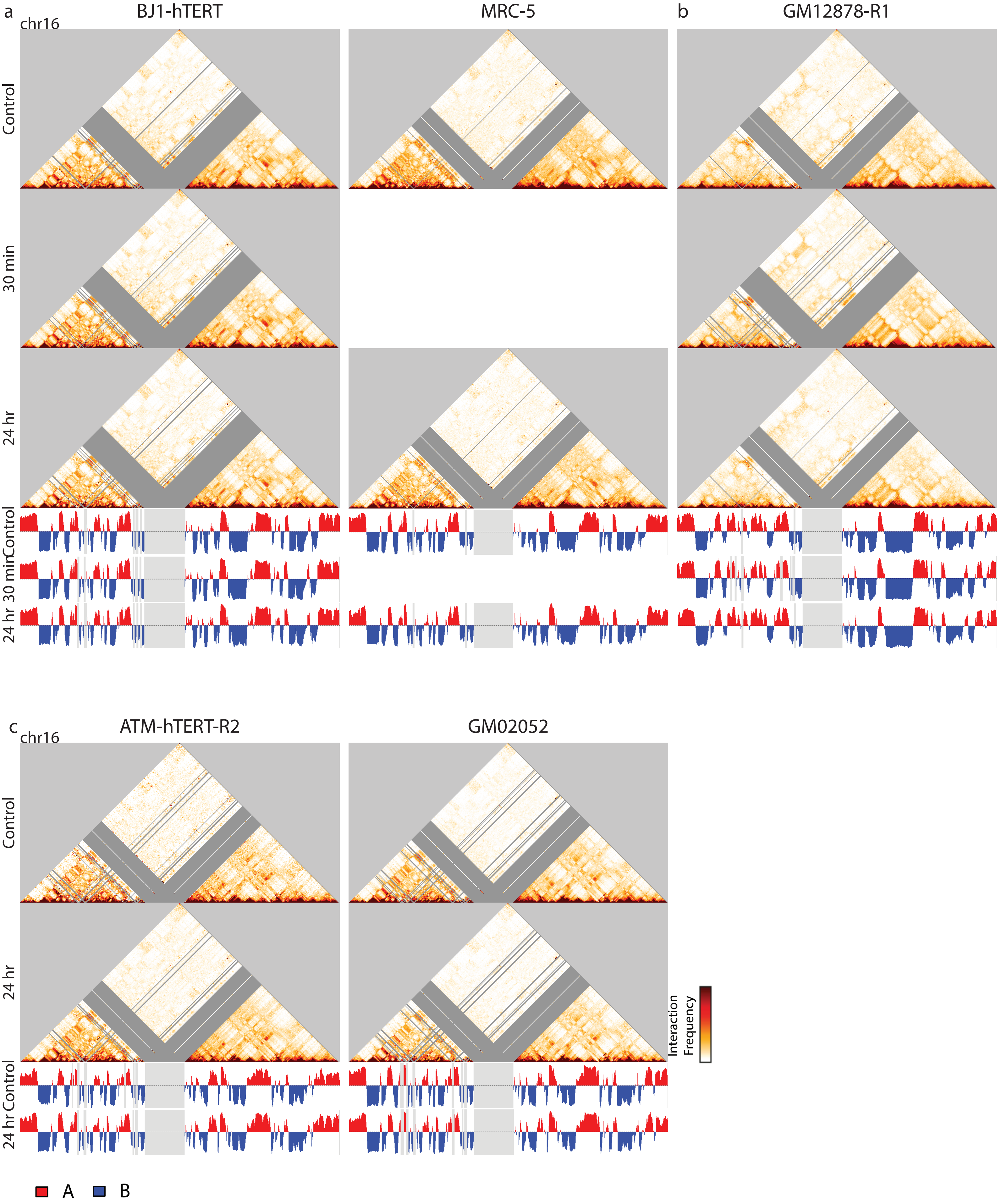

Supplementary Figure 9. A/B compartment identity is robust to changes post-IR in healthy cell lines and ATM mutant cell lines. **a** 250kb bin size Hi-C interaction heatmaps (top) and plots of the first eigenvector from principal component analysis (bottom) for chromosome 16 in healthy fibroblasts: BJ1-hTERT (left) and MRC-5 (right). **b** 250kb bin size Hi-C interaction heatmaps (top) and plots of the first eigenvector from principal component analysis (bottom) for chromosome 16 in healthy lymphoblastoids: GM12878 Replicate 1. **c** 250kb bin size Hi-C interaction heatmaps (top) and plots of the first eigenvector from principal component analysis (bottom) for chromosome 16 in ATM mutant fibroblasts: ATM-hTERT Replicate 2 (left) and GM02052 (right).

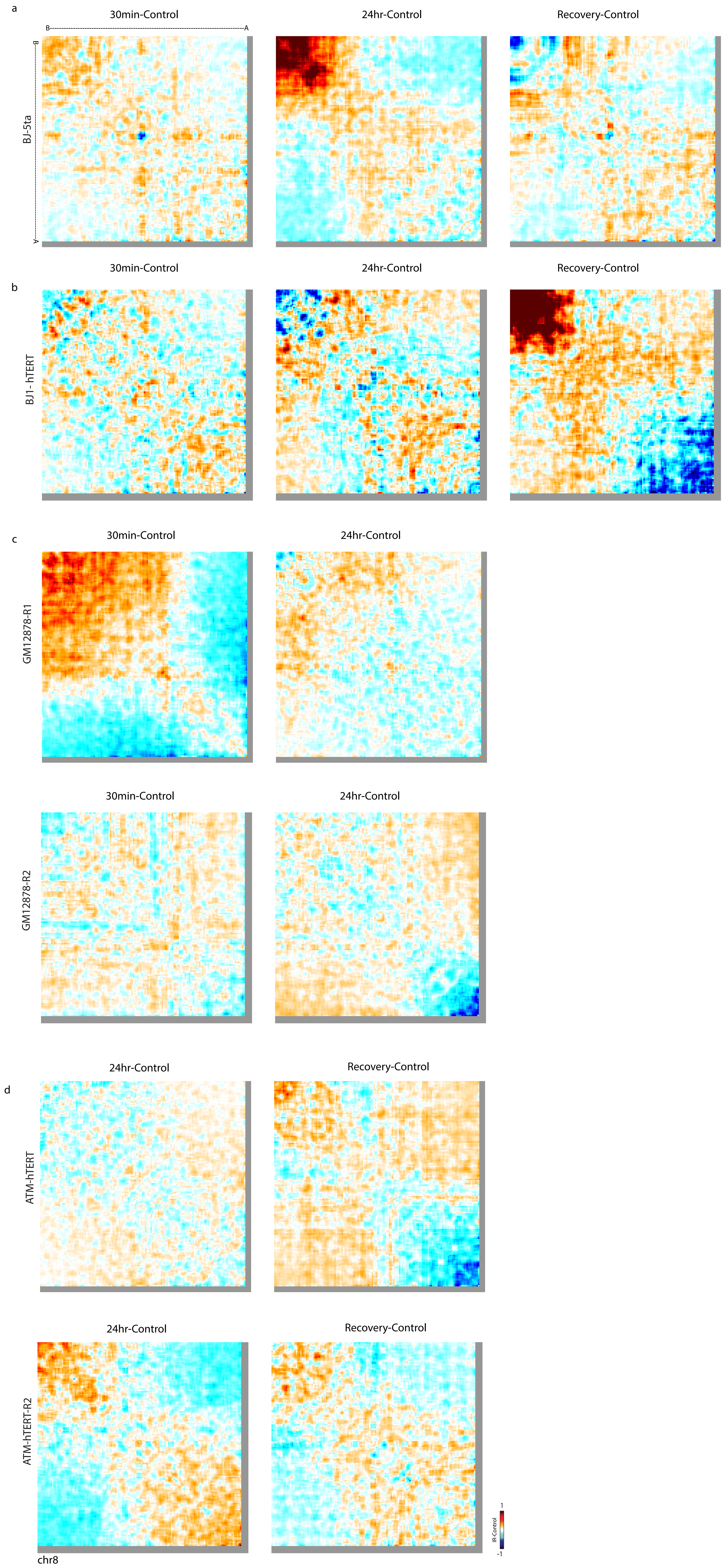

Supplementary Figure 10. Compartment strength changes are subtle and inconsistent between replicates after X-ray. Subtracted saddle plots (showing interaction Z-score differences from Control ordered from strongest B compartment to strongest A compartment, binned and smoothed at 500 kb) for **(a)** BJ-5ta 30 minutes (left), 24 hours (middle), Recovery (right) **(b)** BJ1-hTERT 30 minutes (left) and 24 hours (middle) **(c)** GM12878-R1 (top) and GM12878-R2 (bottom) 30 minutes (left) and 24 hours (middle) **(d)** ATM-hTERT-R1 (top) and ATMhTERT-R2 (bottom) 24 hours (left) and Recovery (middle).

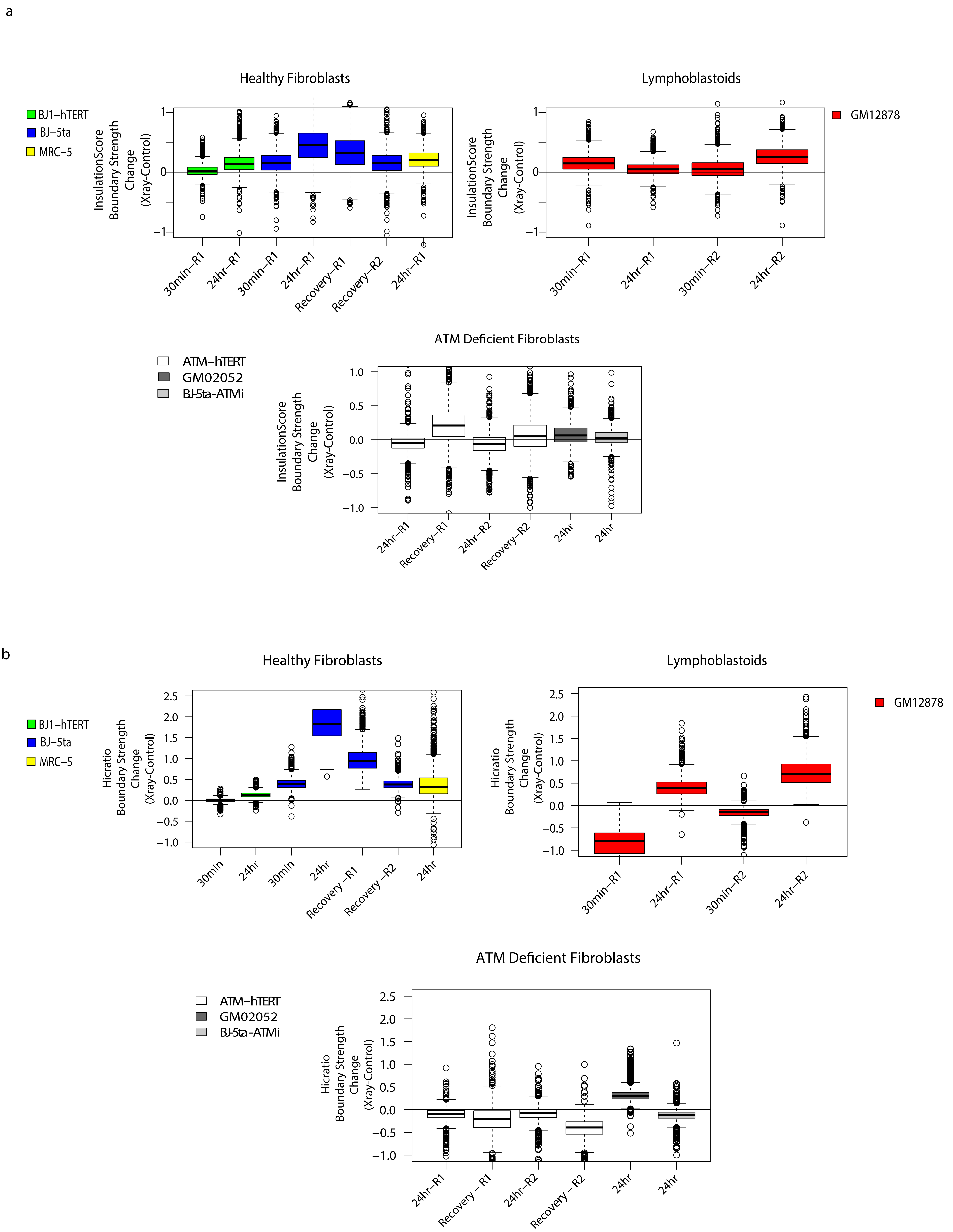

Supplementary Figure 11. TAD boundary strength changes after exposure to IR are consistent between replicates and among similar cell lines. **a** The change in strength for each TAD boundary shared between each cell type's control and IR condition was calculated by InsulationScore method and the distribution of changes are represented in boxplots for all replicates of all cell lines used in the study. Healthy fibroblast lines: BJ1-hTERT (green), BJ-5ta (blue), and MRC-5 (yellow); Lymphoblastoid line: GM12878 (red); ATM deficient fibroblast lines: ATM-hTERT (white), GM02052 (dark gray), BJ5-ta + ATM inhibitor (light gray). Boxes represent the upper and lower quartiles with the center line as the median. Upper whiskers extend 1.5×IQR beyond the upper quartile, and lower whiskers extend either 1.5×IQR below the lower quartile or to the end of the dataset (Number of TAD boundaries (N) BJ-5ta = 1232, BJ1-hTERT = 1310, MRC-5 = 1869, GM12878-R1 = 1371, GM12878-R2 = 1091, ATM-hTERT -R1= 1502, ATM-hTERT-R2 = 1311, GM02052 = 2693, BJ-5ta-ATMi = 2010). **b** Boxplots of changes in TAD boundary strength relative to pre-IR calculated by Hicratio method for all replicates of all cell lines used in study. Healthy fibroblast lines: BJ1-hTERT (green), BJ-5ta (blue), and MRC-5 (yellow); Lymphoblastoid line: GM12878 (red); ATM mutant fibroblast lines: ATM- hTERT (white) and GM02052 (gray). Boxes represent the upper and lower quartiles with the center line as the median. Upper whiskers extend 1.5×IQR beyond the upper quartile, and lower whiskers extend either 1.5×IQR below the lower quartile or to the end of the dataset (Number of TAD boundaries (N) BJ-5ta = 1298, BJ1-hTERT = 1661, MRC-5 = 2471, GM12878-R1 = 1371, GM12878-R2 = 1878, ATM-hTERT -R1= 1971, ATM-hTERT-R2 = 1674, GM02052 = 3258, BJ-5ta-ATMi = 2868).

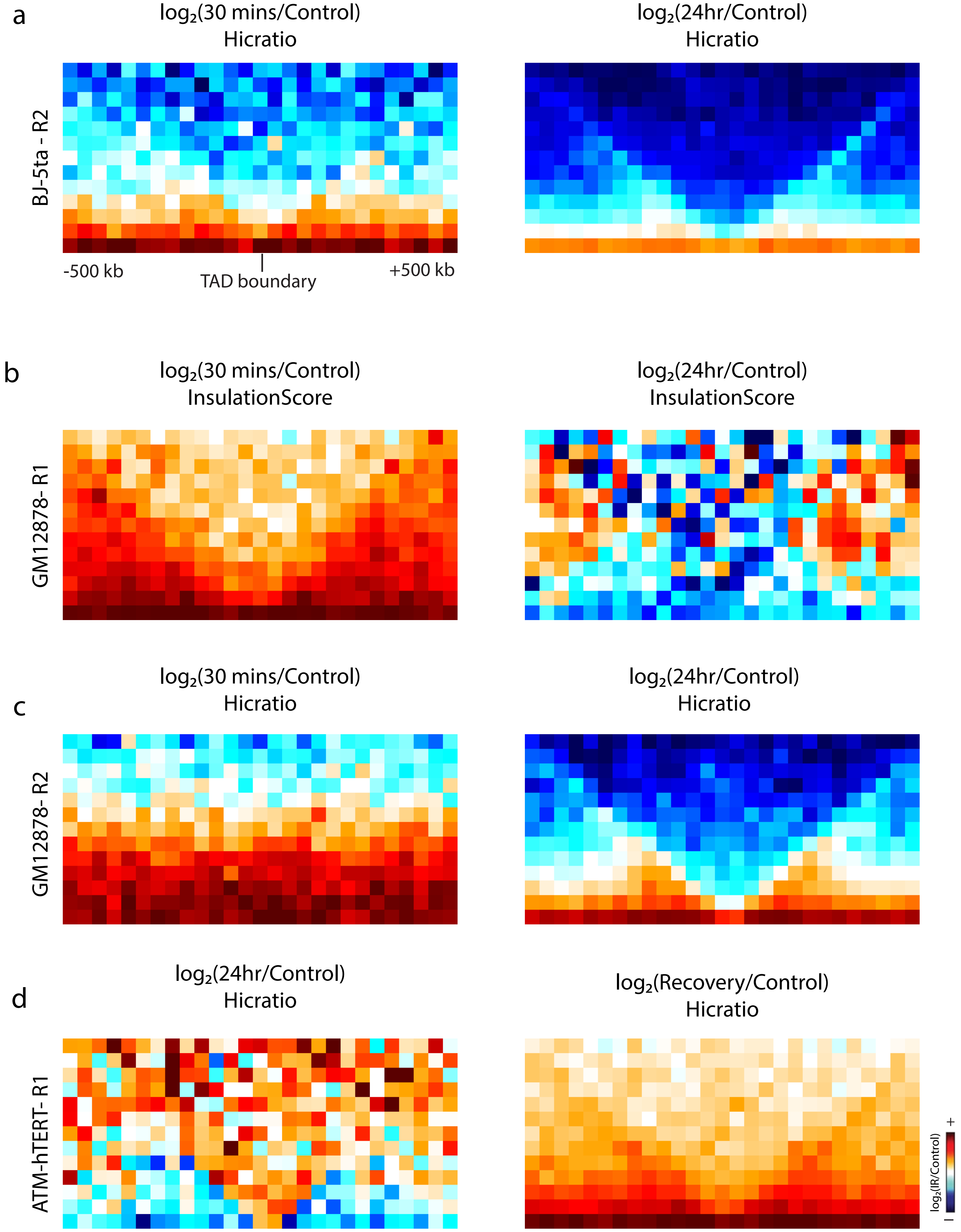

Supplementary Figure 12. Average interactions across TAD boundaries detected using InsulationScore or Hicratio approaches. Aggregate contact maps of averaged TAD boundaries called by Hicratio method in **a** BJ-5ta, **c** GM12878, and **d** ATM-hTERT. **b** Aggregate contact map of averaged TAD boundaries called by InsulationScore method in GM12878-R1 (other replicate not represented in main figure).

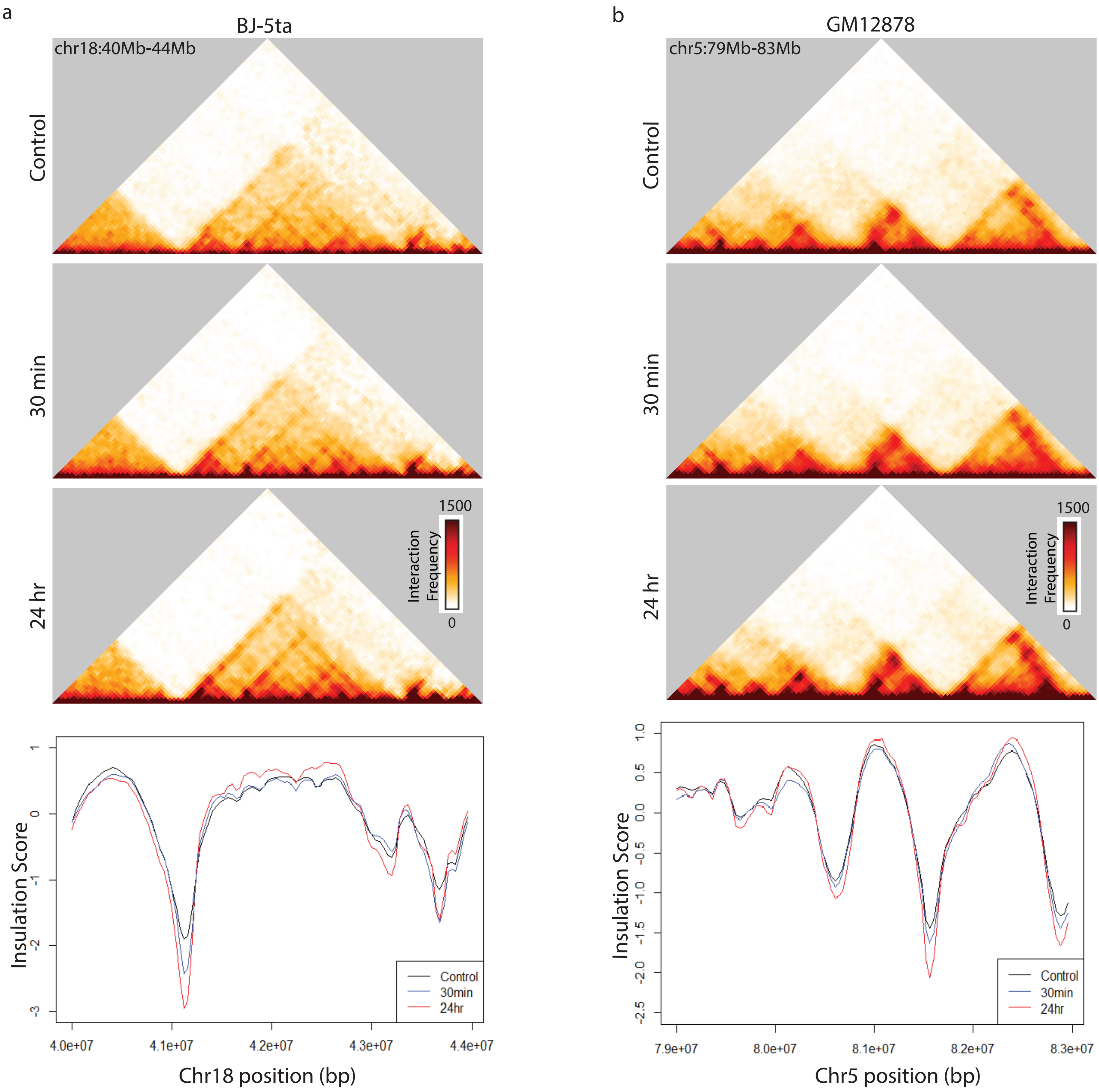

Supplementary Figure 13. Examples of TAD boundary increase. **a** 40 kb heatmaps of BJ-5ta control (top), 30 minutes (middle), and 24 hours (bottom). InsulationScore line plot shows the increase in boundary strength (visible as a decrease in interactions calculated by the insulationScore) between samples. **b** 40 kb heatmaps of GM12878 control (top), 30 minutes (middle), and 24 hours (bottom).

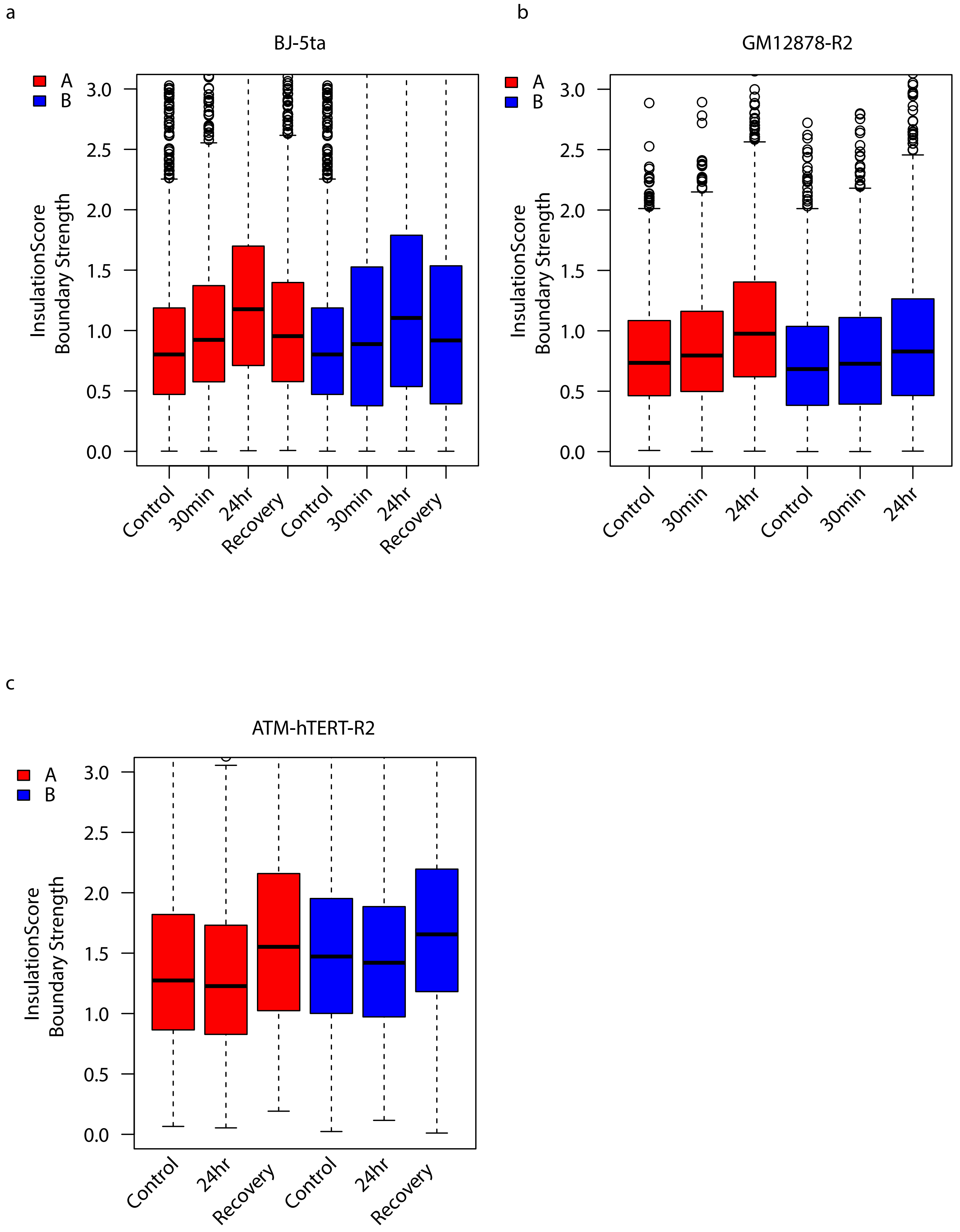

Supplementary Figure 14. TAD boundary strength changes are consistent in both A and B compartments. TAD boundary strength distribution calculated by InsulationScore method for A (red) and B (blue) compartment regions separately for **(a)** BJ-5ta (Number of TAD boundaries (N) BJ-5ta A compartment = 493, B compartment = 434), **(b)** GM12878 (Number of TAD boundaries (N) ATM-hTERT A compartment = 462, B compartment = 496), and **(c)** ATM-hTERT (Number of TAD boundaries (N) ATM-hTERT A compartment = 705, B compartment = 637). Boxes represent the upper and lower quartiles with the center line as the median. Upper whiskers extend 1.5×IQR beyond the upper quartile, and lower whiskers extend either 1.5×IQR below the lower quartile or to the end of the dataset.

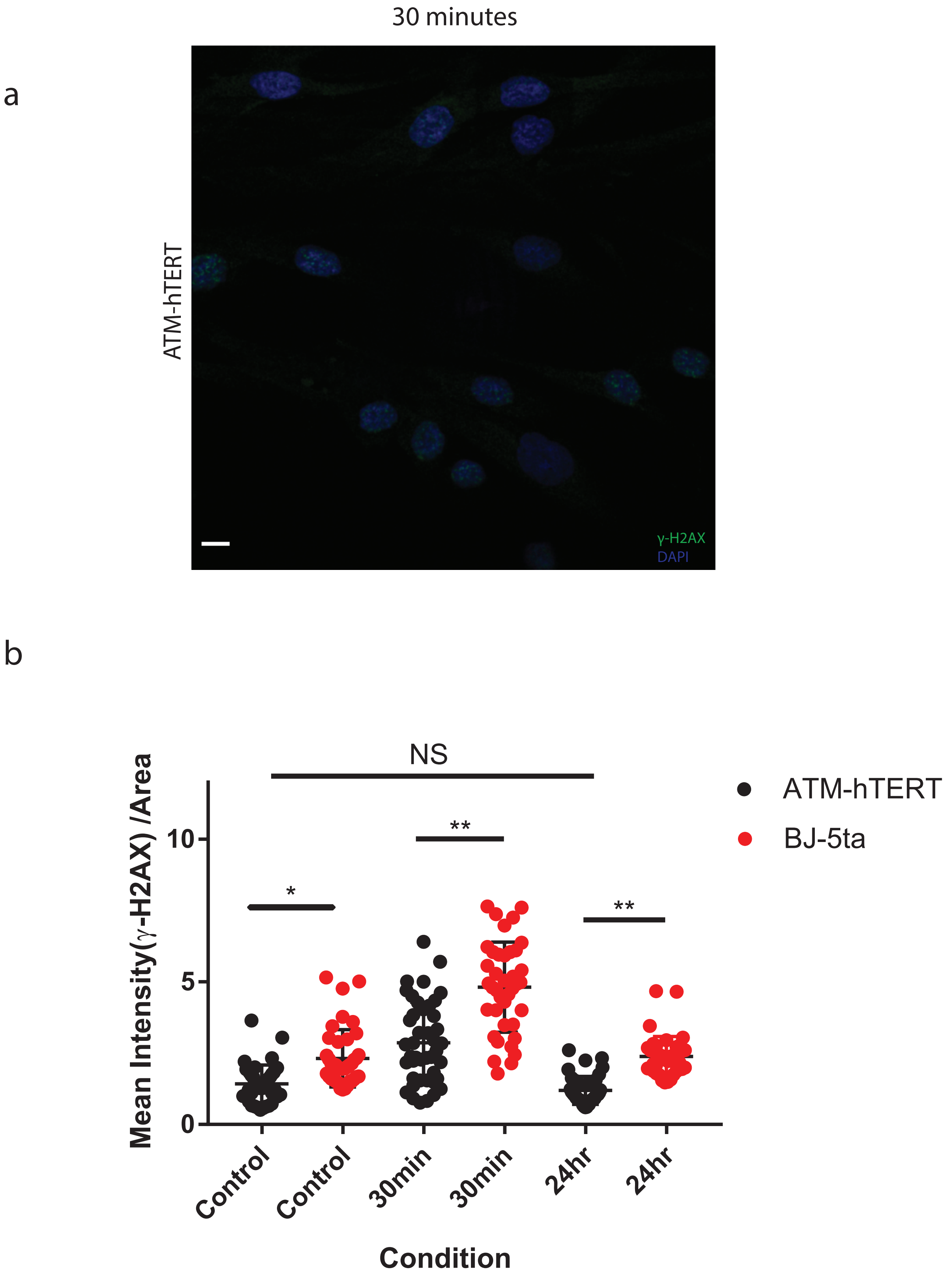

Supplementary Figure 15. γH2AX levels increase in ATM-hTERT cells, but have a weaker response than BJ-5ta. **a** ATM-hTERT fibroblasts stained with γH2AX (green) and DAPI (blue) 30 minutes after exposure to 5 Gy X-rays. Scale bar: 10µm. **b** Quantification of mean fluorescence intensity in ATM-hTERT compared to BJ-5ta data in Supplementary Figure 2a (n = 40 cells, * p <0.0020, ** p<0.0001, one way ANOVA with Tukey's multiple comparison test). γH2AX levels are significantly lower in ATM mutants than BJ-5ta but maintain a similar pattern of increase at 30 mins (p = <0.0001) and return to initial levels at 24hrs (p = ns).

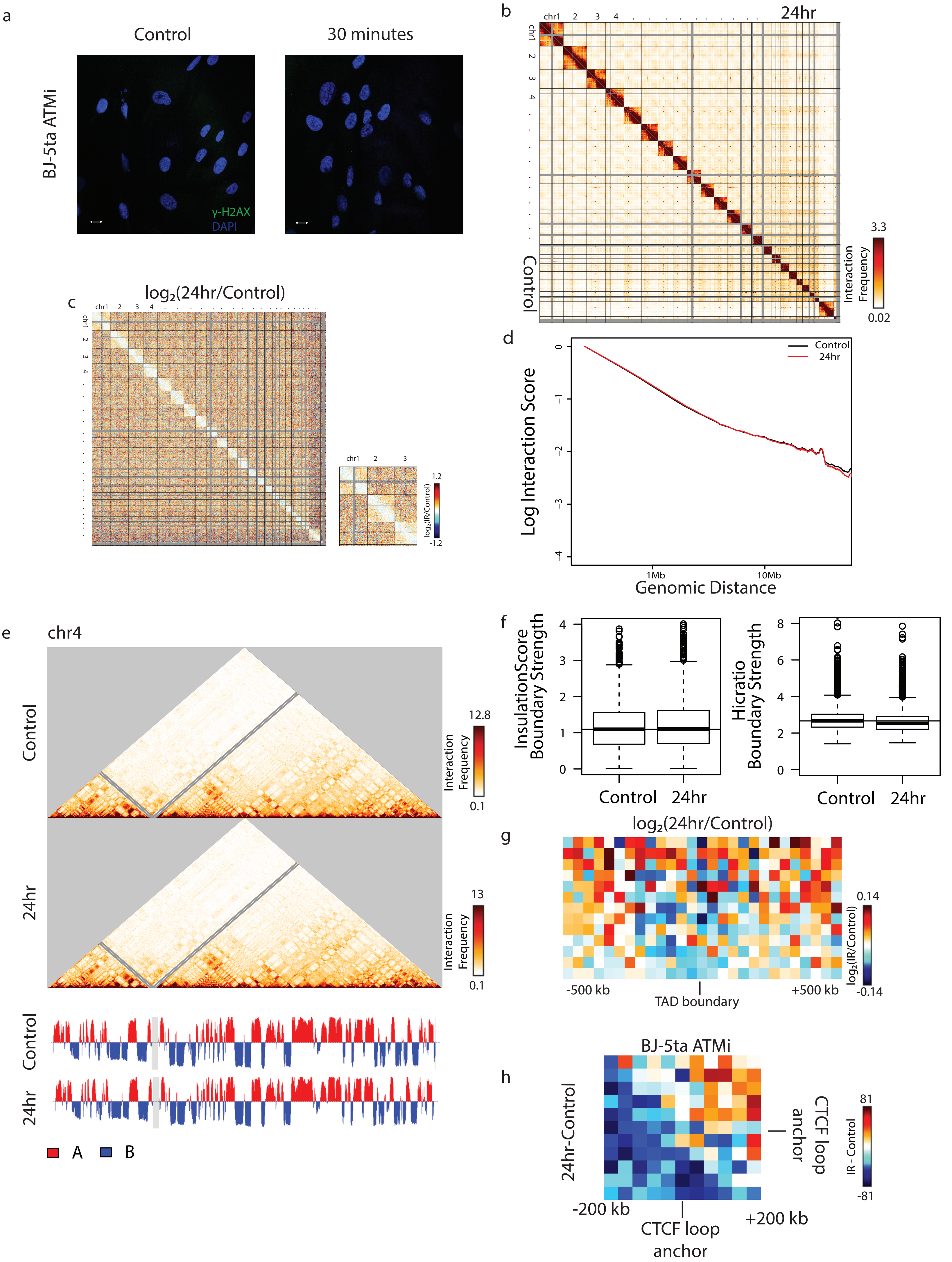

Supplementary Figure 16. ATMi-treated BJ-5ta fibroblasts mimic the effects of ATM deficient patient fibroblasts. **a** ATMi treated BJ-5ta fibroblasts stained with γH2AX (green) and DAPI (blue) before and after exposure to 5 Gy X-rays. Scale bar: 10 µm. **b** Genome wide contact heatmap of ATMi treated BJ-5ta fibroblasts in non-irradiated (bottom left) or 24 h after IR (top right) in 2.5 Mb bins. **c** log2(24 hours post IR/Control) contact heatmap in 2.5 Mb bins. **d** Scaling plots showing average decay of contacts with distance across all chromosomes at a 250 kb bin size in ATMi treated BJ-5ta fibroblasts for control and 24 hours post IR. **e** 250kb bin size Hi-C interaction heatmaps for chromosome 4 in ATMi treated BJ-5ta fibroblasts in control (Top) and 24 hours post IR (Bottom) show little difference in compartment as evidenced by little change to A (red) and B (blue) compartmentalization in the first principle component tracks below heatmaps. **f** TAD boundary strength boxplots calculated using both the InsulationScore (Number of TAD boundaries (N) BJ-5ta-ATMi = 2010). and Hicratio (Number of TAD boundaries (N) BJ-5ta-ATMi = 2868) methods for ATMi treated BJ-5ta fibroblasts. A one-tailed Wilcoxon signed rank test showed that the 24 hour sample boundaries were not statistically increased compared to controls. **g** Contact maps were aggregated at TAD boundaries called by InsulationScore with strength greater than 1 and then these maps are compared by log2(24 h post IR/Control). **h** Subtraction of 24 hours minus Control aggregate CTCF-anchored loop contact maps in ATMi treated BJ-5ta fibroblasts. Boxes represent the upper and lower quartiles with the center line as the median. Upper whiskers extend 1.5×IQR beyond the upper quartile, and lower whiskers extend either 1.5×IQR below the lower quartile or to the end of the dataset.

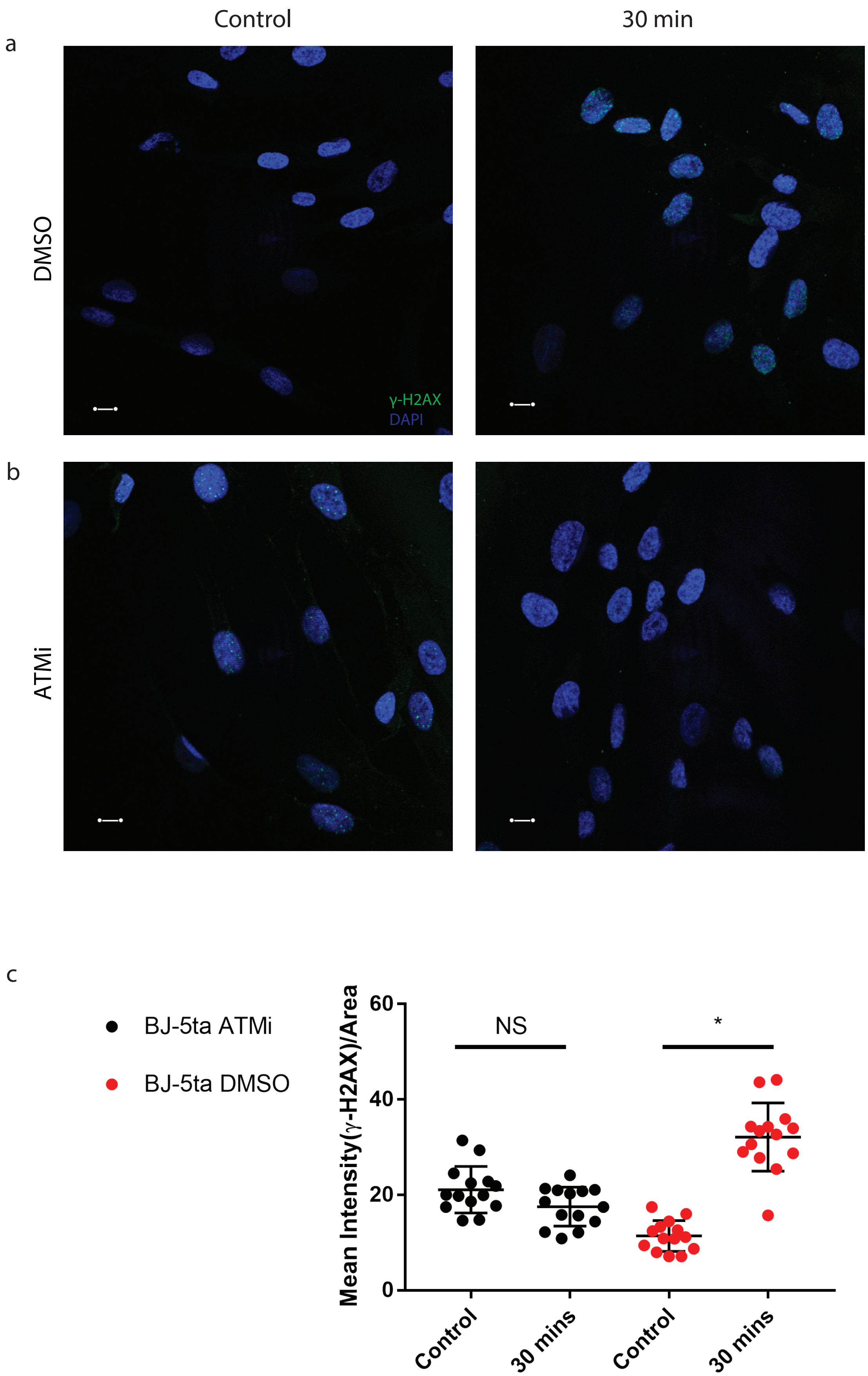

Supplementary Figure 17. ATMi results in decreased γH2AX foci. BJ-5ta fibroblasts were exposed to **(a)** 10µM DMSO or **(b)** 10 µM KU-55933 for one hour and then irradiated with 5 Gy X-rays and imaged at 30 minutes after IR. Each were stained with γH2AX (green) and DAPI (blue) after before or after exposure to 5 Gy X-rays. Scale bar: 10µm. **c** Quantification of ATMi or DMSO treated BJ-5ta fibroblasts mean fluorescence intensity in BJ-5ta cells before and after exposure to IR (n=14 cells, * $p < 0.0001$, one way ANOVA with Tukey's multiple comparison test. Data is presented as the mean of SD with the 25th (bottom line) and 75th (Top line) percentile shown).

Cell Line	Condition	Replicate	Raw # Reads	# Both Sides Mapped	Valid Pairs	Unique Valid Pairs	% Cis	Dangling Ends %
BJ-5ta	Control	R1	180,650,273	112,234,560	98,769,926	62,194,681	74.4	10.3
BJ-5ta	30 mins X	R1	172,469,514	105,572,234	101,168,488	76,520,452	77.9	3.8
BJ-5ta	24 hr X	R1	439,993,397	294,611,193	285,966,444	212,551,030	86.2	1.7
BJ-5ta	Recovery	R1	387,242,758	247,317,471	242,377,386	141,227,429	86.2	1.3
BJ-5ta	Recovery	R2	283,878,060	173,354,500	165,142,153	104,333,341	77.6	4.3
BJ-1 hTERT	Control	R1	246,979,150	178,487,837	166,441,060	129,865,596	84.4	6.2
BJ-1 hTERT	30 min X	R1	231,840,475	168,157,410	155,628,339	125,629,148	83.8	6.9
BJ-1 hTERT	24 hr X	R1	205,760,134	147,470,034	134,110,303	109,198,540	84.8	8.4
ATM-hTERT	Control	R1	204,830,725	168,107,250	141,109,197	124,465,656	78	15.8
ATM-hTERT	Recovery	R1	389,931,788	255,700,038	243,088,728	125,064,421	87.4	4.0
ATM-hTERT	24 hr X	R1	179,977,928	149,541,854	121,284,841	111,110,533	78.6	18.5
ATM-hTERT	Control	R2	60,818,985	41,342,146	34,216,181	31,768,043	76.9	16.9
ATM-hTERT	24 hr X	R2	198,588,290	138,204,859	110,691,110	102,849,684	77.8	19.5
ATM-hTERT	Recovery	R2	98,687,970	63,318,090	59,208,561	48,238,983	82.9	6.0
GM12878	Control	R1	169,041,926	141,427,409	111,380,425	95,881,352	77.7	20.9
GM12878	30 min X	R1	404,673,351	254,353,026	248,835,472	175,309,206	80	1.4
GM12878	24 hr X	R1	192,011,866	157,657,922	122,355,226	107,876,878	75.2	22.0
GM12878	Control	R2	198,416,626	141,010,973	138,034,618	124,040,375	77.4	1.7
GM12878	30 mins X	R2	242,425,056	156,489,034	146,862,286	115,111,914	79.9	5.8
GM12878	24 hr X	R2	190,188,740	136,357,434	131,529,834	106,197,889	83	2.6
MRC5	Control	R1	128,868,956	105,055,075	90,160,337	66,947,233	73.1	14.0
MRC5	24 hr X	R1	95,372,972	77,336,363	52,976,419	43,945,126	82.7	31.3
GM02052	Control	R1	402,772,517	254,020,052	224,921,471	154,220,231	83.7	10.8
GM02052	24 hr X	R1	384,201,796	247,283,552	221,226,346	139,824,607	84.7	9.8
BJ-5ta-ATMi	Control	R1	203,499,867	169,635,019	156,651,916	54,517,589	81.2	7.3
BJ-5ta-ATMi	24 hr X	R1	191,845,870	159,361,684	147,495,534	50,917,138	78.8	7.1

Supplementary Table 1. Mapping statistics for Hi-C samples.

Cell Line	Medium	FBS Content	Additional Supplements
BJ-5ta	4:1 DMEM:Medium 199	10%	Hygromycin B (0.01 mg/mL)
BJ1-hTERT	4:1 DMEM:Medium 199	10%	Hygromycin B (0.01 mg/mL)
GM12878	RPMI 1640	15%	
MRC-5	MEM	10%	
AG04405 (ATM-hTERT)	DMEM	15%	1X Non-essential amino acids
GM02052	MEM	10%	1X Non-essential amino acids

Supplementary Table 2. Cell culture media recipes.
