# Supplementary material for: Radiation-induced DNA damage and repair effects on 3D genome organization
Source: Nat Commun. 2020 Dec 2;11:6178. doi: 10.1038/s41467-020-20047-w (PMC7710719; doi:10.1038/s41467-020-20047-w)
Supplement: Supplementary file 2 — Reporting Summary [file 41467_2020_20047_MOESM2_ESM.pdf]

nature research

Corresponding author(s): Rachel Patton McCord

Last updated by author(s): 9/21/2020

Reporting Summary

Nature Research wishes to improve the reproducibility of the work that we publish. This form provides structure for consistency and transparency in reporting. For further information on Nature Research policies, see Authors & Referees and the Editorial Policy Checklist.

Statistics

For all statistical analyses, confirm that the following items are present in the figure legend, table legend, main text, or Methods section.

n/a	Confirmed	
☐	☒	The exact sample size (*n*) for each experimental group/condition, given as a discrete number and unit of measurement
☐	☒	A statement on whether measurements were taken from distinct samples or whether the same sample was measured repeatedly
☐	☒	The statistical test(s) used AND whether they are one- or two-sided<br>*Only common tests should be described solely by name; describe more complex techniques in the Methods section.*
☐	☒	A description of all covariates tested
☐	☒	A description of any assumptions or corrections, such as tests of normality and adjustment for multiple comparisons
☐	☒	A full description of the statistical parameters including central tendency (e.g. means) or other basic estimates (e.g. regression coefficient) AND variation (e.g. standard deviation) or associated estimates of uncertainty (e.g. confidence intervals)
☐	☒	For null hypothesis testing, the test statistic (e.g. *F*, *t*, *r*) with confidence intervals, effect sizes, degrees of freedom and *P* value noted<br>*Give P values as exact values whenever suitable.*
☒	☐	For Bayesian analysis, information on the choice of priors and Markov chain Monte Carlo settings
☒	☐	For hierarchical and complex designs, identification of the appropriate level for tests and full reporting of outcomes
☐	☒	Estimates of effect sizes (e.g. Cohen's *d*, Pearson's *r*), indicating how they were calculated

Our web collection on statistics for biologists contains articles on many of the points above.

Software and code

Policy information about availability of computer code

Data collection	Attune Nxt V 2.1 was used for flow cytometry data collection.
Data analysis	Hi-C mapping and iterative correction scripts are available through the github cMapping pipeline. Hi-C analysis scripts are available through the cworld-dekker pipeline version 1.01 available on github. Cell cycle analysis was done using Flowing Software 2.5.1, Hicratio and reproducibility calculations were performed with custom scripts that are available on github: https://github.com/rpmccordlab/XrayHiCAnalysis. ImageJ version 1.51i was used for average fluorescence intensity calculations.

For manuscripts utilizing custom algorithms or software that are central to the research but not yet described in published literature, software must be made available to editors/reviewers. We strongly encourage code deposition in a community repository (e.g. GitHub). See the Nature Research guidelines for submitting code & software for further information.

Data

Policy information about availability of data

All manuscripts must include a data availability statement. This statement should provide the following information, where applicable:

- Accession codes, unique identifiers, or web links for publicly available datasets
- A list of figures that have associated raw data
- A description of any restrictions on data availability

All relevant data supporting the key findings of this study are available within the article and its Supplementary Information files or from the corresponding author on reasonable request. The Hi-C data generated in this study have been deposited in Gene Expression Omnibus (GEO) under accession number GSE136899. A source data file is provided with the manuscript.

Field-specific reporting

Please select the one below that is the best fit for your research. If you are not sure, read the appropriate sections before making your selection.

☒ Life sciences ☐ Behavioural & social sciences ☐ Ecological, evolutionary & environmental sciences

For a reference copy of the document with all sections, see nature.com/documents/nr-reporting-summary-flat.pdf

Life sciences study design

All studies must disclose on these points even when the disclosure is negative.

Sample size	The sample size for each Hi-C experiment ranged from ~5-20 million cells, as suggested in accepted protocols such as Golloshi et al. Methods, 2018 (10.1016/j.ymeth.2018.04.033). This sample size allowed sufficient library complexity for good dynamic range in contact counts. The sample size for each microscopy experiment was 15-40 nuclei per condition which provided us sufficient power to detect differences in DNA damage marker intensity. Western blots were loaded with at least 10 micrograms, consistent with previously published experimental protocols.
Data exclusions	Hi-C experiments with dangling end percentages above 35% were excluded due to excessive potential noise and loss of valid pair data. One BJ5ta Hi-C replicate experiment was also excluded due to excessive local inward facing pairs, leading to concerns about digestion efficiency.
Replication	Each Hi-C experiment was repeated twice with different biological samples that were obtained on different dates at various passages. The only exceptions were for BJ1-hTERT (which were limited in availability and reproduced results of very similar BJ5ta cells), MRC-5 (which were only used to confirm the consistent effects observed in BJ cells in a different cell type) and ATMi (which served to confirm results from ATM mutant cells). Microscopy experiments for Supp Fig 2, 6, and 15 included two replicates with 20 cells per condition measured for each replicate. Each experiment included Control, 30 minutes, and 24 hours. Two biological replicates obtained on different dates and with various passages was used for each condition. Microscopy experiments for Supp Fig 3 and 17 included one replicate (at least 15 nuclei each measured). Western blots were repeated twice with consistent results.
Randomization	Groups of cells were separated into experimental conditions of exposure or lack of exposure to X-rays. The cell populations were identical before they were split into X-ray and control groups, preventing any systematic bias in group assignment.
Blinding	Blinding was not necessary for this study because all samples were processed in parallel with identical treatments, and clear labeling of conditions was necessary to avoid mis-assignment of results.

Reporting for specific materials, systems and methods

We require information from authors about some types of materials, experimental systems and methods used in many studies. Here, indicate whether each material, system or method listed is relevant to your study. If you are not sure if a list item applies to your research, read the appropriate section before selecting a response.

Materials & experimental systems

n/a	Involved in the study	
☐	☒	Antibodies
☐	☒	Eukaryotic cell lines
☒	☐	Palaeontology
☒	☐	Animals and other organisms
☒	☐	Human research participants
☒	☐	Clinical data

Methods

n/a	Involved in the study	
☒	☐	ChIP-seq
☐	☒	Flow cytometry
☒	☐	MRI-based neuroimaging

Antibodies

Antibodies used	Alexa Fluor 488 goat anti-mouse (Invitrogen R37120) goat anti-rabbit IRDye 680RD (Licor 92568071), goat anti-mouse IRDye 800CW (Licor 95-32210), γ-H2AX (mouse monoclonal [9F3], Abcam), Beta actin (rabbit polyclonal PA1-16889, Thermo Fisher or mouse monoclonal MA1-140, Thermo Fisher)
Validation	Abcam: Our stringent quality control and validation processes use a variety of techniques, including western blot, ICC/IF, IHC, flow cytometry, ELISA, ChIP, IP and peptide array. The team thoroughly analyze the results obtained in our labs to guarantee the quality of our products and that all information is accurate and available to our customers.<br>Gao S et al. LncRNA LCPAT1 is involved in DNA damage induced by CSE. Biochem Biophys Res Commun 508:512-515 (2019).<br>Plappert-Helbig U et al. Gamma-H2AX immunofluorescence for the detection of tissue-specific genotoxicity in vivo. Environ Mol Mutagen 60:4-16 (2019).<br><br>ThermoScientific: Invitrogen antibodies that have been verified using independent antibodies are indicated with a "verified specificity" symbol in search results and on relevant product pages. The data showing the verification will be provided on each

product page.
Salati S et al. Deregulated expression of miR-29a-3p, miR-494-3p and miR-660-5p affects sensitivity to tyrosine kinase inhibitors in CML leukemic stem cells. Oncotarget ;8(30):49451-49469 (2017)
Liu et al. CRISPR/Cas9-mediated hypoxia inducible factor-1α knockout enhances the antitumor effect of transarterial embolization in hepatocellular carcinoma. Oncology Reports40(5):2547-2557 (2018)

Eukaryotic cell lines

Policy information about cell lines

Cell line source(s)
BJ-5ta (ATCC), MRC-5 (ATCC), GM12878 (Coriell Institute), GM02052 (Coriell Institute), AG04405 (Coriell Institute), BJ-1 hTERT (Clontech)

Authentication
Cells were used directly after purchase from ATCC or Coriell; no authentication was performed in our laboratory.

Mycoplasma contamination
MRC-5, BJ-1 hTERT, GM12878, and AG04405 were tested for mycoplasma by use of PCR with primers designed to detect mycoplasma contamination. All cell lines tested negative for mycoplasma contamination. Primers were derived from Uphoff CC, Drexler HG. (2004) Detecting Mycoplasma contamination in cell cultures by polymerase chain reaction. Methods Mol Med. 88: 319-326. and validated using a positive control. BJ-5ta and GM02052 were purchased freshly from respective institutions.

Commonly misidentified lines
(See ICLAC register)
No commonly misidentified cell lines were used in the study

Flow Cytometry

Plots

Confirm that:

- [x] The axis labels state the marker and fluorochrome used (e.g. CD4-FITC).
- [x] The axis scales are clearly visible. Include numbers along axes only for bottom left plot of group (a 'group' is an analysis of identical markers).
- [x] All plots are contour plots with outliers or pseudocolor plots.
- [x] A numerical value for number of cells or percentage (with statistics) is provided.

Methodology

Sample preparation
Cells were fixed before or after irradiation and fixed with 70% ethanol for 30 minutes at 4°C. Cells were then centrifuged for 10 minutes at 800×g for 10 minutes and resuspended with Guava® Cell Cycle Reagent (Luminex, 4500-0220).

Instrument
Attune Nxt Acoustic Focusing Cytometer

Software
Attune Nxt software version 2.1 and Flowing Software 2.5.1

Cell population abundance
Cells were only analyzed, not sorted

Gating strategy
The detailed gating strategy is described in Supplementary Figure 1b. Initial cell population gating was manually applied on FSC v SSC to exclude evident debris. Then this same gate was placed on the scatter plot for the blue detector vs. SSC to eliminate doublets.
Single cell gating was then plotted in a histogram and cell cycle phases were determined manually, followed by the software's Gaussian fit function. The GM gating of cell cycle phases was transposed to all other conditions for quantitation.

- [x] Tick this box to confirm that a figure exemplifying the gating strategy is provided in the Supplementary Information.
